# A novel prediction model for survival in individual patients with cardiac resynchronization therapy with a defibrillator: Analysis of the new Japan cardiac device treatment registry database

**DOI:** 10.1002/joa3.13213

**Published:** 2025-01-07

**Authors:** Hisashi Yokoshiki, Akihiko Shimizu, Takeshi Mitsuhashi, Kohei Ishibashi, Tomoyuki Kabutoya, Yasuhiro Yoshiga, Yusuke Kondo, Taro Temma, Masahiko Takagi, Hiroshi Tada, Members of the Implantable Cardioverter‐Defibrillator (ICD) Committee of the Japanese Heart Rhythm Society

**Affiliations:** ^1^ Department of Cardiovascular Medicine Sapporo City General Hospital Sapporo Japan; ^2^ Ube Kohsan Central Hospital Ube Japan; ^3^ Department of Cardiovascular Medicine Hoshi General Hospital Koriyama Japan; ^4^ Department of Cardiovascular Medicine National Cerebral and Cardiovascular Center Suita Japan; ^5^ Division of Cardiovascular Medicine, Department of Medicine Jichi Medical University School of Medicine Shimotsuke Japan; ^6^ Division of Cardiology, Department of Medicine and Clinical Science Yamaguchi University Graduate School of Medicine Yamaguchi Japan; ^7^ Department of Cardiovascular Medicine Chiba University Graduate School of Medicine Chiba Japan; ^8^ Department of Cardiovascular Medicine Hokkaido University Hospital Sapporo Japan; ^9^ Division of Cardiac Arrhythmia Kansai Medical University Medical Centre Moriguchi Japan; ^10^ Department of Cardiovascular Medicine, Faculty of Medical Sciences University of Fukui Fukui Japan

**Keywords:** cardiac resynchronization therapy with a defibrillator (CRT‐D), prediction model, survival probability, systolic heart failure, the new Japan cardiac device treatment registry database (new JCDTR)

## Abstract

**Background:**

Accurate prediction for survival in individualized patients with cardiac resynchronization therapy with a defibrillator (CRT‐D) is difficult.

**Methods:**

We analyzed the New Japan cardiac device treatment registry (JCDTR) database to develop a survival prediction model for CRT‐D recipients.

**Results:**

Four hundred and eighty‐two CRT‐D recipients, at the implantation year 2018–2021, with a QRS width ≥120 ms and left ventricular ejection fraction (LVEF) ≤35% at baseline, were analyzed. During an average follow‐up of 21 ± 10 months, death occurred in 66 of 482 CRT‐D patients (14%). A prediction model estimating annual survival probability was developed using Cox regression with internal validation. With seven explanation predictors (age >75 years, serum creatinine >1.4 mg/dL, blood hemoglobin <12 g/dL, heart rate ≥90/min, LVEF, prior NSVT, and QRS width <150 ms), the model distinguished patients with and without all‐cause death, with an optimism‐corrected C‐statistics of 0.766, 0.764, and 0.768, and calibration slope of 1.01, 1.00, and 1.00 at 1 year, 2 years, and 3 years. Additionally, we have devised the calculator of survival probability for individual CRT‐D recipients.

**Conclusions:**

Using routine available variables, we have developed a survival prediction model for individual CRT‐D recipients.

## Graphical abstract

With routine available clinical variables, we have developed a calculator for the individualized survival probability in contemporary CRT‐D recipients with good discrimination and excellent calibration performance.

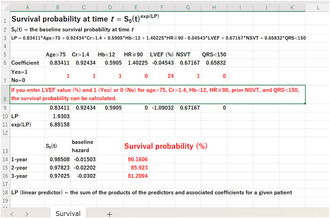

## 1 INTRODUCTION

Patients with heart failure generally are symptomatic for some time before receiving the diagnosis. With initiation of appropriate treatment and fluid management, the heart failure symptoms may diminish promptly at the early stage.^1^ In this regard, ambulatory patients with heart failure are likely to substantially overestimate their life expectancy.^2^

Symptomatic heart failure patients with a left ventricular ejection fraction (LVEF) of 35% or less and QRS width of 120 ms or more can benefit from cardiac resynchronization therapy (CRT).^3^, ^4^, ^5^ Such patients can be at high risk of life‐threatening ventricular tachyarrhythmias, thereby receiving a defibrillator (CRT‐D) to prevent sudden cardiac death.^4^, ^6^ On the other hand, progressive heart failure and non‐arrhythmic modes of death appear to dominate in those with frailty and accumulating comorbidity.^7^ However, model‐based survival predictions for patients with CRT‐D are scarce especially in recent years.^8^, ^9^, ^10^

The present study is aimed to develop a prediction model for survival in individual patients receiving CRT‐D. To this end, we assessed outcomes of the latest CRT‐D cohort from the new Japan cardiac device treatment registry (New JCDTR) database.

## 2 METHODS

### 2.1 Study population

The Japan cardiac device treatment registry (JCDTR) and new JCDTR were established in 2006 and 2018, respectively, by the Japanese Heart Rhythm Society (JHRS) for a survey of actual conditions in patients undergoing de novo implantation of cardiac implantable electronic devices (CIEDs) including implantable cardioverter‐defibrillator (ICD)/cardiac resynchronization therapy with a defibrillator (CRT‐D)/cardiac resynchronization therapy with a pacemaker (CRT‐P).^11^, ^12^, ^13^, ^14^ A new system, called New JCDTR 2023, was started in April 2023, in which data of patients at the implantation date after April 2023 are encouraged to be registered (https://new.jhrs.or.jp/contents_web/new_jcdtr/, accessed on September 2, 2024). The protocol for the research project has been approved by a suitably constituted Ethics Committee at each institution and it conforms to the provisions of the Declaration of Helsinki.

Six hundred and ten consecutive CRT‐D patients registered in the New JCDTR, with the implant date from January 2018 to October 2021, were analyzed in a previous study, in which the device programming and study outcomes were given.^15^ Among them, 47 patients were excluded for QRS width less than 120 msec at baseline. We also excluded 13 patients with missing data on the level of blood hemoglobin or serum creatinine, which are considered to be independent variables for mortality.^8^, ^9^ After that, 69 patients were excluded for LVEF more than 35%. Finally, 482 CRT‐D recipients were included in this study.

### 2.2 Statistical analysis

All data are expressed as mean ± SD. Simple between‐group analysis was conducted using Student's *t*‐test. Categorical variables were compared using the *χ* ^2^ test or Fisher's exact test. Kaplan–Meier curves were constructed to estimate event‐free outcomes in the study groups. Differences with *p* < .05 were considered significant. Statview version 5.0 for Windows (SAS Institute Inc., Cary, NC, USA) or EZR version 1.64 (https://www.jichi.ac.jp/saitama‐sct/SaitamaHP.files/statmedEN.html, accessed on January 28, 2024)^16^ was used for all statistical analyses. We installed “rms” package in the EZR for regression modeling strategy (https://www.youtube.com/watch?v=xPSK5uDjxSs, accessed on May 5, 2024).

### 2.3 Model development and validation

We aimed to develop an individualized survival prediction model using data from 482 CRT‐D recipients of the New JCDTR (as stated above) in accordance with Transparent Reporting of a multivariable prediction model for Individual Prognosis Or Diagnosis (TRIPOD) statement.^17^

To obtain predictors for all‐cause mortality, a multivariate Cox proportional‐hazards regression model was used after obtaining variables that reached a significance level of *p* < .1 in univariate models. Except for LVEF, numeric variables were made binary by the use of cut‐off points with receiver operating characteristic (ROC) curves. The final model was fitted using stepwise backward selection based on Akaike's Information Criterion. For individual patients, the survival probability was calculated using the following equation:

$$\text{Survival probability}\;\mathrm{at}\;\text{time}\;t=S_{0}{\left(t\right)}^{\exp\left(\mathrm{LP}\right)}$$

where S_0_(*t*) is the baseline survival probability at time *t* (e.g., S_0_ (*3*) is the baseline survival probability at 3 years), and linear predictor (LP) is the sum of the product of the predictors and associated coefficients for a given patient. We took the LP as a prognostic index for each patient.^18^ The discriminative performance of the model was measured using “Survival ROC” function with Harrell's C‐statistic. The model was validated using 500 bootstrap samples. The degree of optimism was estimated by the average calibration slope of the bootstrap samples using “rms” package. Agreement between predicted and observed outcomes was graphically evaluated with the calibration plots.

## 3 RESULTS

### 3.1 Outcomes and characteristics of CRT‐D recipients in the new JCDTR

**FIGURE 1:**
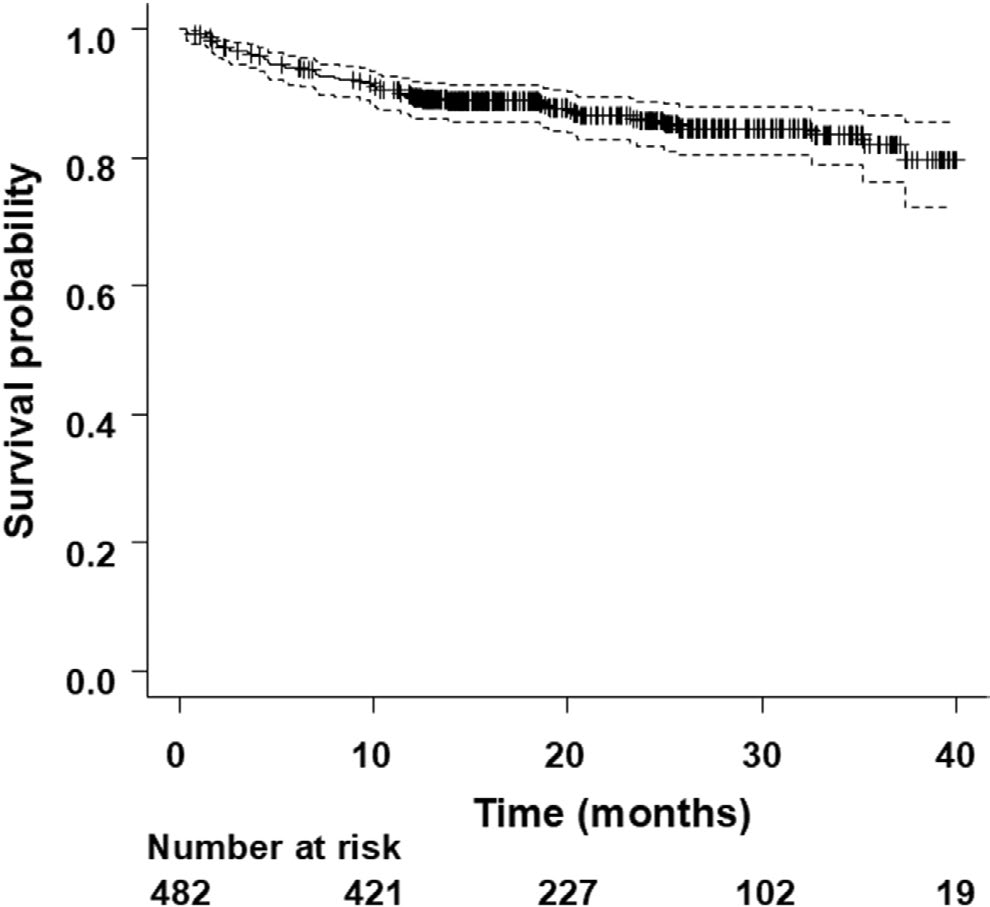
Cumulative survival free from all‐cause death in CRT‐D recipients of the New JCDTR. Cumulative survival probability is plotted with 95% confidence intervals (dotted lines).

All‐cause death occurred in 66 of 482 CRT‐D patients (14%) during an average follow‐up of 21 ± 10 months. The cause of death was heart failure in 34 patients (52%), sudden in 12 patients (18%), other cardiac death in 5 patients (8%), and non‐cardiac in 15 patients (23%). Overall, the survival rate was 89.6% at 1‐year (95% confidence interval [CI]: 86.4–92.0), 85.7% at 2‐year (95% CI: 81.7–88.8), and 82.1% at 3‐year (95% CI: 76.3–86.5) (Figure 1).

Patients with death had a higher age, lower body weight, and lower body mass index compared to those without death. The rate of primary prevention for sudden cardiac death was 65.2% and 76.7% in patients with and without death (*p* = .044). LVEF was lower, and heart rate was higher in patients with death than those without death. QRS width and New York Heart Association (NYHA) classification tended to be shorter and more severe in patients with death, although there was no significant difference between the two groups. The rate of prior non‐sustained ventricular tachycardia (NSVT) was 72.7% and 58.7% in patients with and without death (*p* = .030). The level of blood hemoglobin (Hb) was lower and that of serum creatinine (Cr) was higher in patients with death than those without death. Other clinical characteristics and pharmacological therapies of study population are summarized in Table 1 and Table 2.

**TABLE 1:** Characteristics of the patients.

	Overall (*n* = 482)	Patients without death (*n* = 416)	Patients with death (*n* = 66)	*p* value
Age (years)	68.5 ± 10.8	68.2 ± 10.6	70.7 ± 11.9	.072
Male	369 (76.6)	318 (76.4)	51 (77.3)	.88
Height (cm)	162 ± 9	162 ± 10	162 ± 8	.95
Body weight (kg)	60 ± 14	61 ± 14	56 ± 11	.0096
Body mass index (kg/m^2^)	22.9 ± 4.8	23.1 ± 4.9	21.4 ± 3.9	.0057
Primary prevention	362 (75.1)	319 (76.7)	43 (65.2)	.044
Underlying heart disease				.51
Ischemic	130 (27.0)	110 (26.4)	20 (30.3)	
Non‐ischemic	352 (73.0)	306 (73.6)	46 (69.7)	
LVEF (%)	25.3 ± 6.3	25.6 ± 6.2	23.5 ± 6.4	.011
NYHA class				.12
I	14 (2.9)	13 (3.1)	1 (1.5)	
II	176 (36.5)	157 (37.7)	19 (28.8)	
III	256 (53.1)	219 (52.6)	37 (56.1)	
IV	36 (7.5)	27 (6.5)	9 (13.6)	
Heart rate (/min)	70 ± 16	69 ± 15	74 ± 18	.019
QRS duration (ms)	161.8 ± 25.9	162.6 ± 25.7	156.6 ± 26.4	.078
QT interval (ms)	472.7 ± 51.9	475.0 ± 51.4	458.7 ± 53.6	.018
Cardio‐thoracic ratio (%)	57.3 ± 6.5	57.1 ± 6.4	58.7 ± 6.9	.066
Atrial lead				.72
Absent	42 (8.7)	37 (8.9)	5 (7.6)	
Present	440 (91.3)	379 (91.1)	61 (92.4)	
NSVT	292 (60.6)	244 (58.7)	48 (72.7)	.030
AF	164 (34.0)	138 (33.2)	26 (39.4)	.32
Diabetes mellitus	168 (34.9)	139 (33.4)	29 (43.9)	.096
Hypertension	222 (46.1)	195 (46.9)	27 (40.9)	.37
Dyslipidemia	216 (44.8)	182 (43.8)	34 (51.5)	.24
Hyperuricemia	130 (27.0)	108 (26.0)	22 (33.3)	.21
Cerebral infarction	45 (9.3)	43 (10.3)	2 (3.0)	.058
Peripheral artery disease	22 (4.6)	18 (4.3)	4 (6.1)	.53
Chronic kidney disease	259 (53.7)	212 (51.0)	47 (71.2)	.0022
COPD	21 (4.4)	14 (3.4)	7 (10.6)	.0074
BNP (pg/mL)^a^	747 ± 1048	663 ± 942	1286 ± 1459	<.0001
Hemoglobin (g/dL)	13.0 ± 2.1	13.1 ± 2.1	12.2 ± 2.1	.0010
Creatinine (mg/dL)	1.64 ± 1.61	1.56 ± 1.52	2.19 ± 2.01	.0028

*Note*: Values are means ± SD, or number (%).

^a^ The value of BNP was missed in 92 patients without death and 15 patients with death.

Abbreviations: AF, atrial fibrillation; BNP, B‐type natriuretic peptide; COPD, chronic obstructive pulmonary disease; NSVT, non‐sustained ventricular tachycardia; NYHA, New York Heart Association.

**TABLE 2:** Pharmacological therapy.

	Overall (*n* = 482)	Patients without death (*n* = 416)	Patients with death (*n* = 66)	*p* value
Ia	5 (1.0)	5 (1.2)	0 (0.0)	.37
Ib	12 (2.5)	10 (2.4)	2 (3.0)	.76
Beta blockers	401 (83.2)	350 (84.1)	51 (77.2)	.17
III	211 (43.8)	173 (41.6)	38 (57.6)	.015
Ca^2+^ antagonists	33 (6.8)	29 (6.9)	4 (6.1)	.79
Digitalis	18 (3.7)	17 (4.1)	1 (1.5)	.31
Diuretics	390 (80.9)	335 (80.5)	55 (83.3)	.59
ACEI/ARB	302 (62.7)	270 (64.9)	32 (48.5)	.010
MRA	224 (46.5)	197 (47.4)	27 (40.9)	.33
Nitrates	26 (5.4)	23 (5.5)	3 (4.5)	.74
Statins	206 (42.7)	176 (42.3)	30 (45.5)	.63
SGLT2 inhibitor	42 (8.7)	38 (9.1)	4 (6.1)	.41
Oral anticoagulants	210 (43.6)	171 (41.1)	39 (59.1)	.0062
Antiplatelet drugs	175 (36.3)	148 (35.6)	27 (40.9)	.40

*Note*: Data are given as number (%). Ia, Ib, Ic, and III indicate the class Ia, Ib, Ic, and III antiarrhythmic drugs, respectively.

Abbreviations: ACEI, angiotensin‐converting enzyme inhibitor; ARB, angiotensin II receptor blocker; MRA, mineralocorticoid receptor antagonist; SGLT2, sodium‐glucose cotransporter‐2.

### 3.2 Model development

Table 3 summarizes multivariable predictors for all‐cause death, each of which was obtained using stepwise backward selection and had a significant linear or log‐linear relationship with the outcome. Therefore, all predictors were fitted into a multivariable model. The following formula allows for the calculation of the survival probability at 3‐years:

$$\text{Survival probability}\;\mathrm{at}\;3-\text{years}=0.97025^{\exp\left(\mathrm{LP}\right)}$$

where LP = 0.83411 × Age >75 + 0.92434 × Cr >1.4 + 0.5905 × Hb <12 + 1.40225 × HR ≥90–0.04543 × LVEF + 0.67167 × NSVT + 0.65832 × QRS <150. “0.97025” is the baseline survival probability at 3 years (S_0_ (*3*)), which is the value obtained from exponential function of minus baseline hazard at 3 years. Data S1 provides the baseline survival probability (S_0_(*t*)) at 1, 2, and 3 years and allows for the calculation of survival probability for individual patients.

**TABLE 3:** All‐cause mortality risk prediction in CRT‐D recipients of the New JCDTR.

	Regression coefficient	Hazard ratio	95% CI	*p* value
Age >75 years	0.83411	2.30	1.40–3.80	.001
Creatinine >1.4 mg/dL	0.92434	2.52	1.53–4.15	<.001
Hemoglobin <12 g/dL	0.5905	1.80	1.10–2.97	.020
Heart rate ≥90/min	1.40225	4.06	2.31–7.16	<.001
LVEF (per % increase)	−0.04543	0.96	0.92–0.99	.019
Prior NSVT	0.67167	1.96	1.12–3.41	.018
QRS width <150 ms	0.65832	1.93	1.17–3.19	.010

Abbreviations: CI, confidence interval; LVEF, left ventricular ejection fraction; NSVT, non‐sustained ventricular tachycardia.

**FIGURE 2:**
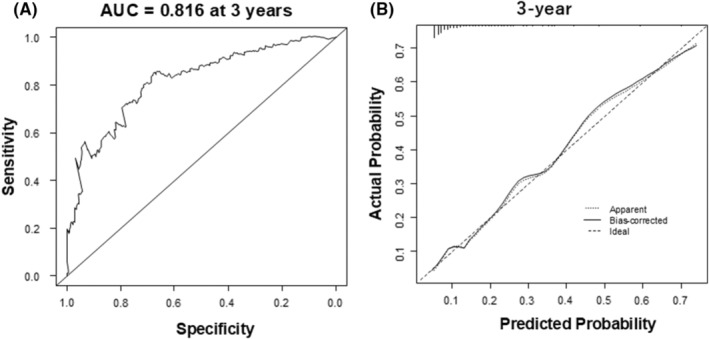
The model discrimination and calibration performance. (A) Time‐dependent receiver operating characteristic (ROC) curve, (B) calibration plot at 3 years showing the agreement between predicted (*x* axis) and observed (*y* axis) risk of the all‐cause death.

The C‐statistics of the predictive model was 0.767 (95% CI 0.701–0.832). With Survival ROC function, it was 0.744, 0.775, and 0.816 at 1 year, 2 years, and 3 years (Figure 2A), respectively.

### 3.3 Model validation

**FIGURE 3:**
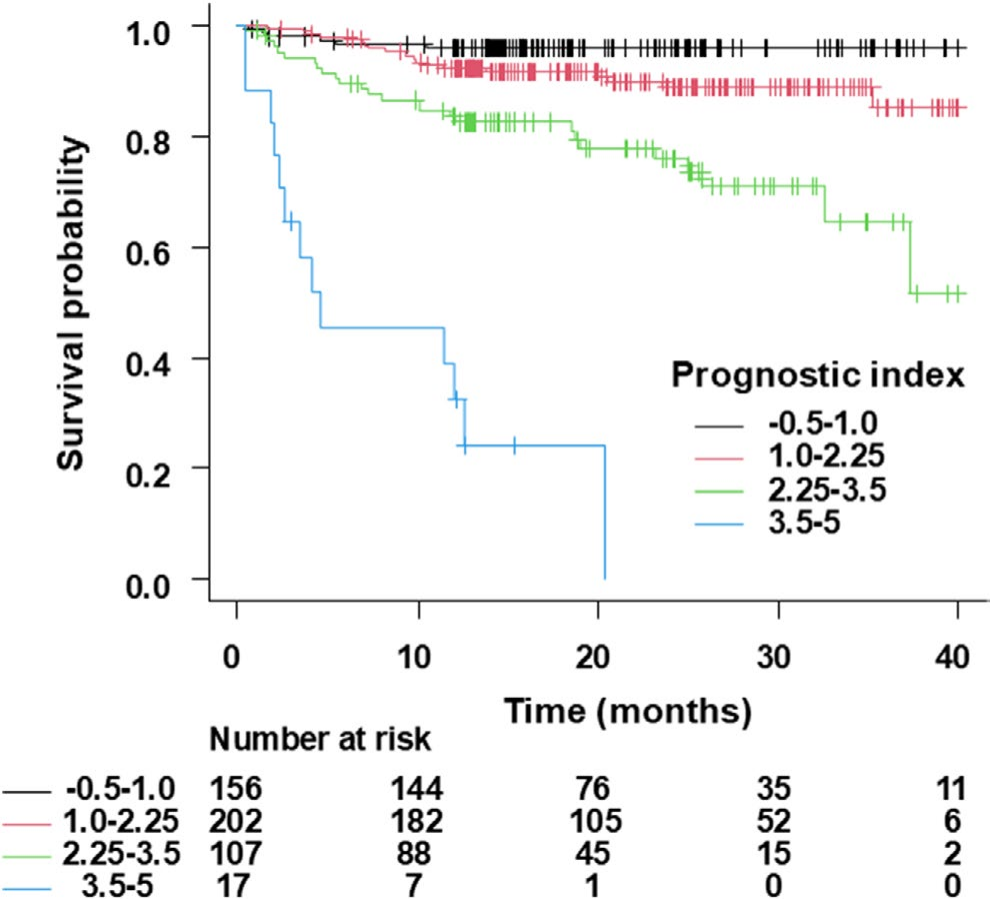
Kaplan–Meier estimates of survival probability in CRT‐D recipients of the New JCDTR stratified by the risk groups, which are classified according to the prognostic index (PI). −0.5 to 1.0 group: −0.5 ≤ PI <1.0; 1.0–2.25 group: 1.0 ≤ PI <2.25; 2.25–3.5 group: 2.25 ≤ PI <3.5; 3.5–5.0 group: 3.5 ≤ PI <5.0.

With 500 bootstrap samples, the optimism‐corrected C‐statistics of the model was 0.766, 0.764, and 0.768 at 1 year, 2 years, and 3 years. The calibration slope of 1.01 (mean absolute error [MAE]: 0.013) at 1 year, 1.00 (MAE: 0.01) at 2 years, and 1.00 (MAE: 0.008) at 3 years (Figure 2B). Optimism of the slope was −0.012, −0.0044, and −0.0097 at 1 year, 2 years, and 3 years, thereby indicating a small degree of optimism without significant overfitting in the predictive model. Figure 3 illustrates the Kaplan–Meier curves for the survival in patients with CRT‐D stratified by the risk groups, which are classified according to the prognostic index. Predicted survival rates were similar to the observed rates (Table 4).

**TABLE 4:** Predicted and observed survival rates in CRT‐D recipients of the different risk groups stratified by the prognostic index (PI).

PI group	At 1 year	At 2 years	At 3 years
−0.5 to 1.0 group (*n* = 156)			
Predicted mean rate (%) (95% CI)	97.6 (97.5–97.7)	96.5 (96.3–96.7)	95.2 (95.0–95.5)
Observed mean rate (%) (95% CI)	96.1 (91.4–98.2)	96.1 (91.4–98.2)	96.1 (91.4–98.2)
1.0–2.25 group (*n* = 202)			
Predicted mean rate (%) (95% CI)	92.7 (92.3–93.0)	89.5 (89.0–89.9)	85.9 (85.2–86.5)
Observed mean rate (%) (95% CI)	92.4 (87.7–95.3)	88.9 (82.9–92.9)	85.2 (74.2–91.8)
2.25–3.5 group (*n* = 107)			
Predicted mean rate (%) (95% CI)	78.4 (77.2–79.6)	70.2 (68.7–71.8)	61.8 (59.9–63.6)
Observed mean rate (%) (95% CI)	83.7 (75.0–89.5)	75.9 (65.1–83.8)	64.5 (46.7–77.7)
3.5–5.0 group (*n* = 17)			
Predicted mean rate (%) (95% CI)	49.8 (44.1–55.6)	36.7 (31.0–42.3)	25.9 (20.9–30.8)
Observed mean rate (%) (95% CI)	32.4 (12.0–54.9)	NA	NA

*Note*: −0.5 to 1.0 group: −0.5 ≤ PI <1.0; 1.0–2.25 group: 1.0 ≤ PI <2.25; 2.25–3.5 group: 2.25 ≤ PI <3.5; 3.5–5.0 group: 3.5 ≤ PI <5.0.

Abbreviations: CI, confidence interval; NA, not applicable; PI, prognostic index.

## 4 DISCUSSION

We have devised a unique calculator (Data S1) for the prediction of individualized survival rates in CRT‐D recipients from the latest cohort of New JCDTR using a multivariable Cox proportional hazard model. It was essential to obtain the baseline survival probability at time t (S_0_(*t*)) for the calculator. The discrimination performance was quasi‐good in terms of the C‐statistics of close to 0.80, and the calibration was excellent with its slope of 1.0. The calculator may guide one's advanced care planning for patients with CRT‐D at the time of the implantation.^19^

Khatib et al. proposed the EAARN score as a predictive score for mortality in CRT recipients.^8^ Among them, the rate of having a CRT‐D was 68%. The EAARN is the acronym for EF <22%, Atrial fibrillation (AF), Age ≥70 years, Renal function (glomerular filtration rate [GFR] <60 mL/min/1.73 m^2^), and baseline NYHA classification IV. Each additional predictor increased the mortality. Nauffal et al. developed the HF‐CRT score with five independent variables including serum high‐sensitivity C‐reactive protein >9.42 ng/L (H), NYHA functional classification III/IV (F), serum creatinine >1.2 mg/dL (C), red blood cell count <4.3 × 10^6^/μL (R), and cardiac troponin T >28 ng/L (T) to predict the composite outcome of all‐cause mortality, left ventricular assist device implant or heart transplantation in primary prevention CRT‐D recipients.^9^ The C‐statistics of the multivariable model was 0.88, indicating a good discrimination. Gasparini et al. designed and validated the VALID‐CRT risk score for all‐cause mortality in CRT recipients using clinical variables including age, gender, LVEF, AF with or without atrioventricular junction ablation, ICD backup, ischemic etiology, NYHA classification III/IV, and diabetes with the C‐statistics of 0.70.^10^

On the other hand, these studies were performed using patients who had undergone CRT implantation before 2013, when the American and European recommendations for CRT implantation were updated with much emphasis on the QRS width and left bundle branch block‐morphology,^20^, ^21^ thereby likely to change CRT population. In fact, there was significant difference with regard to all‐cause mortality between CRT‐D recipients of the JCDTR and New JCDTR.^15^ The difference in study population may lead to spectrum bias^22^, ^23^; thus it would be necessary to develop a new prediction model for the latest CRT‐D recipients.

There are several limitations to be considered in this study. First, the number of patients in the highest‐risk group (3.5–5.0 group) was small and the range of the predicted survival rate was broad (Table 4). Despite this issue, the calibration performance appeared to be acceptable even in the population at the predicted probability of more than .5 (Figure 2B). However, in patients with a prognostic index above 3.5, it may be better to interpret our predictive model with great caution. Second, the number of events (i.e., all‐cause death) was 66, whereas the number of explanation variables was 7, which may cause a problem of overfitting. On the other hand, optimism of the slope with bootstrap validation was small enough to neglect the issue. Third, possible important variables such as serum high‐sensitivity C‐reactive protein and cardiac troponin T are not available in the New JCDTR database. Fourth, it appears to be necessary to differentiate between paroxysmal and persistent AF and to clarify whether catheter ablation for AF was performed, which potentially influences prognosis. However, we do not have the information in the New JCDTR. Fifth, we did not exclude cardiac amyloidosis (4 patients of 482, 0.8%) and cardiac sarcoidosis (43 patients of 482, 9%), which may not respond to standard heart failure treatments. Information with regard to additional therapies like tafamidis or steroids was not available. These limitations highlight the importance of external validation studies in CRT‐D recipients from other populations such as the New JCDTR 2023.

In conclusion, using routinely available clinical variables, we have developed a calculator for the individualized survival probability in contemporary CRT‐D recipients with good discrimination and excellent calibration performance.

## FUNDING INFORMATION

There is no funding source for this study.

## CONFLICT OF INTEREST STATEMENT

The authors declare no conflict of interest.

## ETHICS STATEMENT

The JCDTR and New JCDTR were approved by the Ethics Committee of Sapporo City General Hospital on May 16, 2018 (approval no. H30‐057‐455).

## CONSENT

Patient consent has been obtained in an opt‐out manner in Sapporo City General Hospital.

## Supporting information


Data S1.
